# Unexpected Procedure Difficulties Increasing the Complexity of Transvenous Lead Extraction: The Single Centre Experience with 3721 Procedures

**DOI:** 10.3390/jcm12082811

**Published:** 2023-04-11

**Authors:** Andrzej Kutarski, Wojciech Jacheć, Dorota Nowosielecka, Anna Polewczyk

**Affiliations:** 1 Department of Cardiology, Medical University, 20-059 Lublin, Poland; 2 2nd Department of Cardiology, Faculty of Medical Sciences in Zabrze, Medical University of Silesia in Katowice, 41-800 Zabrze, Poland; 3 Department of Cardiology, The Pope John Paul II Province Hospital, 22-400 Zamość, Poland; 4 Department of Cardiac Surgery, The Pope John Paul II Province Hospital, 22-400 Zamość, Poland; 5 Department of Physiology, Pathophysiology and Clinical Immunology, Institute of Medical Sciences, Jan Kochanowski University, 25-369 Kielce, Poland; 6 Department of Cardiac Surgery, Świętokrzyskie Center of Cardiology, 25-736 Kielce, Poland

**Keywords:** transvenous lead extraction, difficult lead extraction, lead extraction complexity

## Abstract

Background: Transvenous lead extraction (TLE) should be completed, even when facing difficulties which have yet to be described. The aim was to explore unexpected TLE obstacles (the circumstances of the occurrence and influence on TLE outcome). Methods: The retrospective analysis of a single centre database containing 3721 TLEs. Results: Unexpected procedure difficulties (UPDs) occurred in 18.43% of cases (singles in 12.20% of cases and multiples in 6.26% of cases). These included blockages in the lead venous approach in3.28% of cases, functional lead dislodgement in 0.91% of cases, and loss of broken lead fragment in 0.60% of cases. All of them, including implant vein—in 7.98% of cases, lead fracture during extraction—in 3.84% of cases, and lead-to-lead adherence—in 6.59% of cases, Byrd dilator collapse—in 3.41% of cases, including the use of an alternative prolonged the procedure but had no influence on long-term mortality. Most of the occurrences were associated with lead dwell time, younger patient age, lead burden, and poorer procedure effectiveness and complications (common cause). However, some of the problems seemed to be related to cardiac implantable electronic devices (CIED) implantation and the subsequent lead management strategy. A more complete list of all tips and tricks is still required. Conclusions: (1) The complexity of the lead extraction procedure combines both prolonged procedure duration and the occurrence of lesser-known UPDs. (2) UPDs are present in nearly one fifth of the TLE procedures, and can occur simultaneously. (3) UPDs, which usually force the extractor to expand the range of techniques and tools, should become part of the training in transvenous lead extraction.

## 1. Introduction

Transvenous lead extraction (TLE) plays a key role in the management of patients with cardiac implantable electronic devices (CIED). It is a highly effective procedure (over 95%) with an acceptable rate of major complications (1.6–2.5%) [1,2,3,4]. Its goal is to remove the lead in its entirety, with a minimal risk of major complications (depending mainly on the operator’s experience and the utilised tools), and without complication-related deaths (regarding the organization of the procedure) [1,2,3,4]. The procedure has to be successful and completed from start to finish despite the occurrence of unforeseen difficulties (e.g., the fracture of the targeted lead).

The prediction and management of major complications has been the subject of several reports [5,6,7,8]. Increased procedure difficulty was defined as prolonged extraction or fluoroscopy time [9,10,11,12,13,14,15,16], the use of advanced tools and methods [12,13,14,15,16], or as the increased number of laser pulses delivered [10,17]. However, far too little attention has been paid to the prediction of procedure difficulties and increased complexity, as these terms are still poorly defined. Difficult lead extraction refers to the lack of advancement of the sheath (using the implant vein access site), thus forcing the extractor to change the tools according to the principle of the stepwise approach the and cross-over strategy (larger polypropylene sheaths, mechanical rotational threaded tip sheaths or laser sheaths) [18]. As a consequence, the duration of extraction and fluoroscopy use was longer than usual [9,10,11,12,13,14,15,16,17]. However, the occurrence and significance of unforeseen obstacles/procedure difficulties such as blockages in the lead implant vein/subclavian region [19,20,21], polypropylene dilator collapse/fracture [21,22], lead-to-lead adhesion [23,24,25,26,27], lead fracture during extraction [25,26,27,28,29,30,31,32,33,34], the use of an alternative venous approach [16,21,24,25,26,27,28,29,30,31,32,34], the loss of a broken lead fragment [34,35,36,37,38,39], or the dislodgement of functional lead have been described in case reports, but never in larger case series reports. Our database of 3721 extractions prompted us to perform an in-depth analysis of the occurrence and significance of unexpected procedure difficulties (UPDs).

Goal of the study. The aim of this study was to identify unexpected procedure difficulties (UPDs) i.e., intraprocedural obstacles (traps/technical problems), explore the circumstances of their occurrence, determine the rate of UPDs and their influence on TLE outcomes, including short-, mid- and long-term mortality. Furthermore, the study set out to investigate the usefulness of most popular scores for the prediction of difficult and complicated lead extraction.

## 2. Methods

### 2.1. Study Population

All transvenous lead extraction (TLE) procedures were performed between March 2006 and June 2022 at a high-volume centre were reviewed. Patient clinical data, the CIED system, and the history of pacing, information on extracted leads, procedure complexity, efficacy, and outcomes were analysed using our retrospective computerized database. The study population is derived from the TLE database of the reference centre, which was previously described [23,24,25,26,27,28,29,30]. All consecutive transvenous lead extraction (TLE) procedures have been entered into the database. No inclusion and exclusion criteria were applied. The entire population was analysed.

### 2.2. Lead Extraction Procedure

Definitions: Indications for lead extraction, procedure effectiveness, complications and efficacy were established according to the recent guidelines (2009 and 2017 HRS consensus and 2018 EHRA guidelines) [1,2,3,4].

Procedure complexity was expressed as procedure duration, global procedure duration (skin-to-skin), extraction time of all leads (sheath-to-sheath time) and average time of single lead extraction (sheath-to-sheath/number of extracted leads). The need to use second-line tools and advanced tools was another marker of increased procedure complexity [9,10,11,12,13,14,15,16]. This study focuses on unexpected procedure difficulties (UPDs) during lead removal that are technical problems that increased procedure complexity but were not considered complications. These include:

Blockage in the lead implant vein/subclavian region that hinders conventional sheath delivery due to scar tissue or too tight of a space between the clavicle and the first rib as a result of the parasternal implantation of the lead (subclavian crush syndrome) with the necessity of using more aggressive tools [19,20,21,23,24].

**Figure 1:**
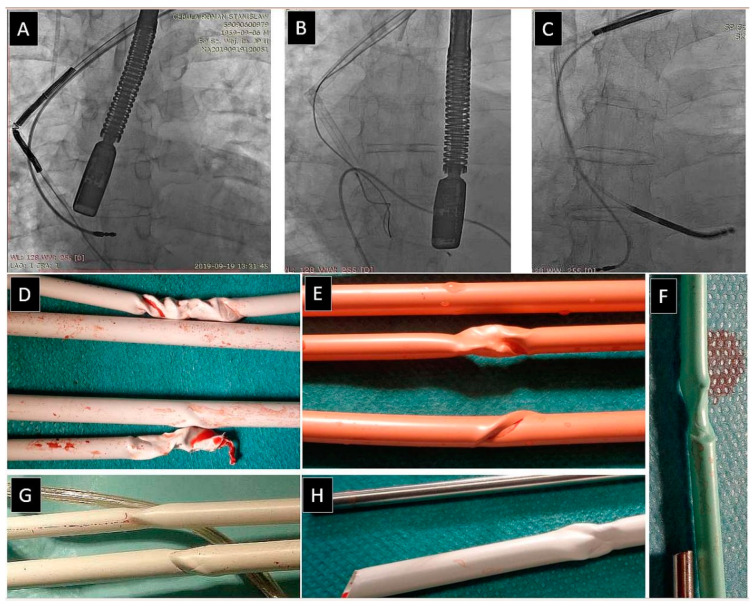
Byrd dilator collapse/fracture. Polypropylene catheter kinking is not a problem if detected early (**A**–**C**). Each successive rotation of the kinked polypropylene catheter rolls it up and tightens on the lead (**E**–**H**), making it more and more difficult to remove (**D**). Good quality fluoroscopy and careful observation of the entire working catheter can prevent secondary major problems [22].

A Byrd dilator collapse/fracture (BDF) can have serious consequences. Generally, there are two types of BDF: (1) An acute angle of sheath deflection that causes fracture, in which the broken sheath is blocked and cannot be moved relative to the lead (to varying degrees); and (2) A delayed BDF when twisting the tube blocks the lead. The sheath can be easily broken into two pieces if pulled or moved forward. BDF (not a complication unless a serious clinical event that may be life-threatening ensues) always requires special and careful sheath manoeuvres (tube removal or shift into the straight segment of the vein), also pulling the lead blocked by the broken sheath fragment, without countertraction, which may cause major procedural complications [21,22] (Figure 1).

Lead-to-lead adherence is when mechanical sheaths fail to disrupt adhesions between two leads, as when one lead is freed, the other lead is pulled down, or is wrapped around the lead being extracted [21,23,24,25,26].

**Figure 2:**
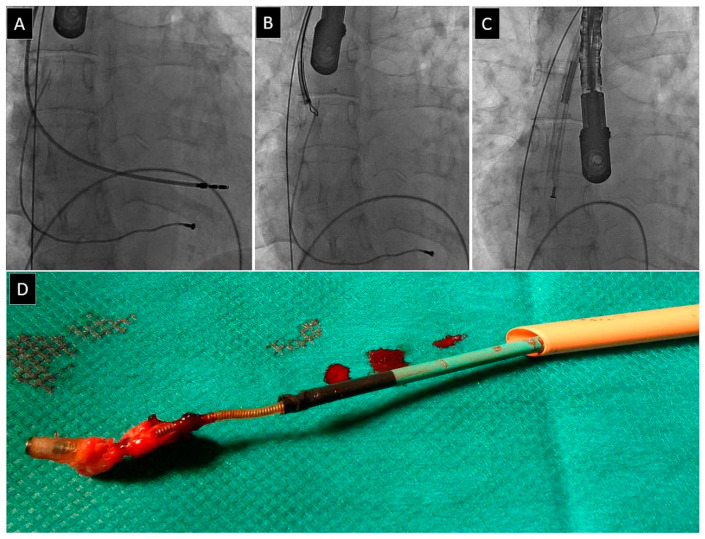
Lead fracture. The broken lead (**A**) can be removed via an inferior or superior approach (using a re-established vein access) (**B**–**D**). The latter technique enables the use of conventional tools for lead dilatation [3].

Lead fracture is defined as the complete break into two parts to be removed separately. The proximal part was easily removed using the dilator sheath. The distal lead part (>4 cm) or fragment (<4 cm) was removed in the next stage of the procedure [21,26,27,28,29,30,31,32,33,34] (Figure 2).

The use of an alternative venous approach refers to all procedures accomplished via a vascular access site other than the implant vein [16,21,24,25,26,28,29,30,31,32,34].

The loss of the broken lead fragment occurs when the main part of the lead is cut free and removed but both ends remain in place and a lead fragment is embolized into the pulmonary vascular bed. This difficulty group also included the loss of the silicone tube in the cardiovascular system. Both were removed in the next stage of the procedure [35,36,37,38,39].

Dislodgement of an active (functional) lead refers to the accidental displacement of the tip of the functional lead or a significant shift of the entire lead that made the venous route inaccessible. Such leads should be replaced, thus increasing the procedure time. We made every effort to avoid the displacement of leads that were difficult to reimplant (left ventricular and His bundle pacing) [40].

### 2.3. Procedure Information

We used a stepwise approach in all patients starting from non-powered mechanical telescoping polypropylene sheaths (Byrd Dilator Sheaths, Cook Medical Inc., USA) of all sizes and lengths, as previously described [23,24,25,26,27,28,29,30]. Laser sheaths were not used. 

In the last 17 years, the organisation of lead extraction has evolved from procedures performed in the electrophysiology laboratory using intravenous analgesia/sedation [8,21] to procedures in the hybrid room in patients only under general anaesthesia. In the last 7 years, the core extraction team has consisted of the same highly experienced extractor (now usually serving as a proctor), experienced echocardiographer, and dedicated cardiac surgeon [21,25,26,27]. A total of 1188 (31.9%) TLE procedures were performed using transoesophageal echocardiographic monitoring, and the cardiac surgeon was the co-operator during 1994 (53.59%) of the procedures. Some 1985 (53.35%) procedures were performed in a hybrid room or in a cardiac surgery operating theatre.

### 2.4. Dataset and Statistical Methods

We split the group into subgroups for the analysis of events and data patients. First, we grouped patients according to the occurrence of obstacles during lead extraction (unexpected procedure difficulties): blockage in the lead implant vein/subclavian region, polypropylene dilator collapse/fracture, lead-on-lead binding, lead fracture, the use of an alternative venous approach, the loss of broken lead fragment or functional lead dislodgement. As an event of interest, unexpected procedure difficulty could occur more than once in the same patient, and the total number of events in our analysis did not correspond to the number of extraction procedures. We analysed potential patient-related risk factors, CIED-related factors, technical problems during lead extraction, TLE complications, efficacy, survival after the procedure, and prognostic factors for the occurrence of UPDs. TLEs involving removal of broken leads with proximal ends in the cardiovascular system were excluded from analysis because the problem had been known before proceeding with the extraction, and therefore such situations did not meet the criteria for an unexpected problem [41].

The statistical significance of the impact of the assessed factors on the occurrence of individual UPDs was determined by comparing the group with a given UPD with the group of patients in whom an event of interest did not occur. All continuous variables are presented as means ± standard deviation. All categorical variables are presented as counts and percentages. Due to unequal sample sizes, the significance of differences was determined using the nonparametric Chi^2^ test with Yates correction or the unpaired Mann-Whitney U test, as appropriate. A linear regression analysis was used to identify predictors of one UDP, more than one UDP, or any UDP occurrence. Variables with *p*-values < 0.05 under univariable analysis were entered into the multivariable models and presented in tables. To determine the impact of UDP on survival, the Kaplan–Meier survival curves were plotted, and were evaluated with the log rank test. A *p*-value less than 0.05 was considered significant. A statistical analysis was performed with Statistica 13.3 (TIBCO Software Inc.).

### 2.5. Approval of the Bioethics Committee

All patients gave their informed written consent to undergo TLE and use anonymous data from their medical records, as approved by the Bioethics Committee at the Regional Chamber of Physicians in Lublin, no. 288/2018/KB/VII. The study was carried out in accordance with the ethical standards of the 1964 Declaration of Helsinki.

## 3. Results

The study population was dominated by non-infectious indications (68.61%), including lead replacement (46.82%), caused by mechanical lead damage (26.93%), lead dysfunction (exit/entry block, extracardiac pacing) (22.55%) and change of pacing mode/upgrading, downgrading (6.26%) (Table 1).

We tried to identify patient-related risk factors for the occurrence of UPD. Table 2 shows that 81.53% of the procedures were trouble-free, one UPD occurred in 12.20% of the procedures, and multiple UPDs occurred in 6.26% of them. The most common UPDs were blockages in the lead implant vein/subclavian region (7.98%) and lead-on-lead binding (6.59%). Other unexpected problems were rare: Byrd dilator collapse/fracture (3.41%), fracture of the targeted lead (3.84%), use of an alternative venous approach (3.28%), functional lead dislodgement (0.91%), and the loss of a broken lead fragment (0.60%). Younger patient age at first system implantation similar to patient age during TLE increased the risk of Byrd dilatator collapse, fracture of the targeted lead, the use of an alternative venous approach and the occurrence of multiple or any UPD. There was no relationship between gender and the occurrence of UPDs. Patients with ischemic heart disease as underlying disease were at a lower risk of most UPDs, especially Byrd dilatator collapse, the use of an alternative venous approach, the loss of a broken lead fragment, and procedures with the occurrence of any technical problem. Higher LVEF values were associated with an increased risk of targeted lead fracture, the use of an alternative venous approach, and multiple UPDs. A low Charlson comorbidity index increased the risk of Byrd dilator collapse/fracture, targeted lead fracture, and the use of an alternative venous approach.

The study shows that the history of CIED and pacing are potential risk factors for increased procedure complexity and major complications. The number of leads in the heart before TLE similar to the presence of 4 and >4 leads in the heart before TLE increased the risk of lead-on-lead binding, the use of an alternative venous approach, the loss of broken lead fragments, and functional lead dislodgement. The presence of abandoned leads before TLE was associated with the more frequent occurrence of UPDs. Leads on both sides of the chest increased the risk of Byrd dilator collapse/fracture, the use of an alternative venous approach, and the loss of a broken lead fragment. The number of procedures before lead extraction in comparison to the other factors had a weak influence on the occurrence of UPDs. The presence of abnormally long lead loops in the atrium or in the ventricle before TLE was often associated with lead-on-lead binding, the fracture of the targeted lead, the use of an alternative venous approach, and the loss of a broken lead fragment. Importantly, in this study all potential risk factors were associated with the more frequent occurrence of multiple UPDs in the same patient.

In the next stage, we checked whether conventional risk factors for major complications of the procedure were also indicators of an increased risk of UPD. And as shown in Table 3, the removal of CIEDs with an ICD lead lowered the probability of UPDs, but the number of extracted leads per patient, similar to extraction of three or more leads, abandoned leads or passive leads, significantly increased the risk of all UPDs; the increase in risk was mainly due to Byrd dilator collapse/fracture, lead-on-lead binding, the fracture of targeted leads, the use of an alternative venous approach, and the loss of a broken lead fragment. It is obvious that the most important risk factor for major complications, namely lead dwell time, was strongly associated with the occurrence of all UPDs (except functional lead dislodgement). To sum up, there are at least four procedure-dependent risk factors for UPDs (the extraction of three or more leads, abandoned lead(s), and passive fixation lead(s), as well as long or very long implant duration).

In the next stage, we examined the relationship between the indicators of TLE complexity (extraction time, use of second-line tools and advanced tools) and the risk of UPD. The most representative indicator of procedure duration (defined by fluoroscopy time) was the extraction time of all leads and the average time of single lead extraction, since global procedure duration includes reimplantation of a new system in non-infectious cases. Thus, the lead extraction time was prolonged for all UPDs (mainly due to the fracture of the targeted lead, the use of an alternative venous approach, and the loss of a broken lead fragment). The occurrence of UPDs was associated with a more frequent use of second-line tools (mechanical rotational threaded tip) or advanced tools (lassos or Dormia basket catheters or other tools for a femoral approach).

The data presented in Table 4 shows that to some extent the predictors of MC also indicate an increased risk of UPDs (SAFeTY, EROS 2, 3). A similar direction of change (but less pronounced) was observed for MB score (the need for advanced tools to achieve TLE success), LED score (difficult TLE defined by fluoroscopy time), and advanced TLE (Mazzone) score (the need for advanced TLE techniques).

It seems that our recently developed [42] lead extraction difficulty score—LECOM score (combined lead extraction time, use of second-line or advanced tools and advanced techniques) is most useful for the prediction of UPDs.

Next, we analysed the relationship between the occurrence of major complications, incomplete lead extraction and procedural success, and the presence of UPDs. Patients with major complications (any), haemopericardium, emergent surgical intervention, and only partial radiographic success (retained lead tip or <4 cm lead fragment) were significantly more likely to have UPDs. These clear differences were not found for tricuspid valve damage during TLE, haemothorax, other major complications because there were too few cases to draw conclusions. Furthermore, similar to previous analyses, it should be underlined that all major complications and partial radiographic success were concurrent with multiple UPDs in the same patient.

There was a relationship between TLE effectiveness expressed as the rate of clinical and procedural success, short-, medium-, and long-term mortality, and the risk of UPDs. Clearly, lower rates of procedural success were noted in patients with UPDs such as Byrd dilator collapse/fracture, lead-on-lead binding, the fracture of the targeted lead, the use of an alternative venous approach, and the loss of a broken lead fragment. These differences were somewhat less pronounced for clinical success; however, they had the same direction of change. This proves a significant impact of these UPDs on the efficiency of TLE. The number of deaths in individual subgroups during the observation period was too small to draw reliable conclusions, but it seems that increased TLE complexity did not influence long-term survival. As previously mentioned the occurrence of multiple UPDs in the same patient significantly reduced the chances of procedural and clinical success (Table 5).

A multivariable regression analysis showed that patient age at first CIED implantation, the number of leads in the heart before TLE, abnormally long lead loops in the atrium or ventricle before TLE, and lead dwell time were independent risk factors for the occurrence of one or multiple UPDs during TLE. Single UPDs were less likely to occur in patients undergoing lead extraction due to CIED infection. Leads on both sides of the chest and the extraction of passive fixation leads were also risk factors for the occurrence of multiple UPDs during TLE (Table 6). 

**Figure 3:**
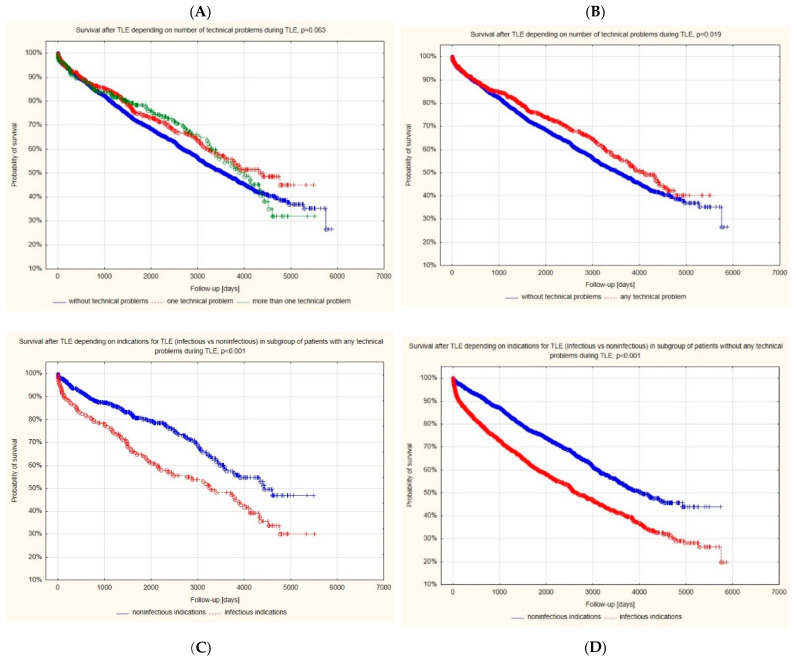
(**A–D**) Prognostic impact of UPDs during lead extraction on survival after TLE.

The Kaplan-Meier survival curves showed that the presence of any UPD during TLE was associated with a better outcome after TLE. This result was probably due to lower patient age during TLE, higher LVEF, a lower Charlson comorbidity index, and a lower percentage of infectious indications for TLE in patients with UPDs during TLE (Figure 3B).

### Results Recapitulation

**Figure 4:**
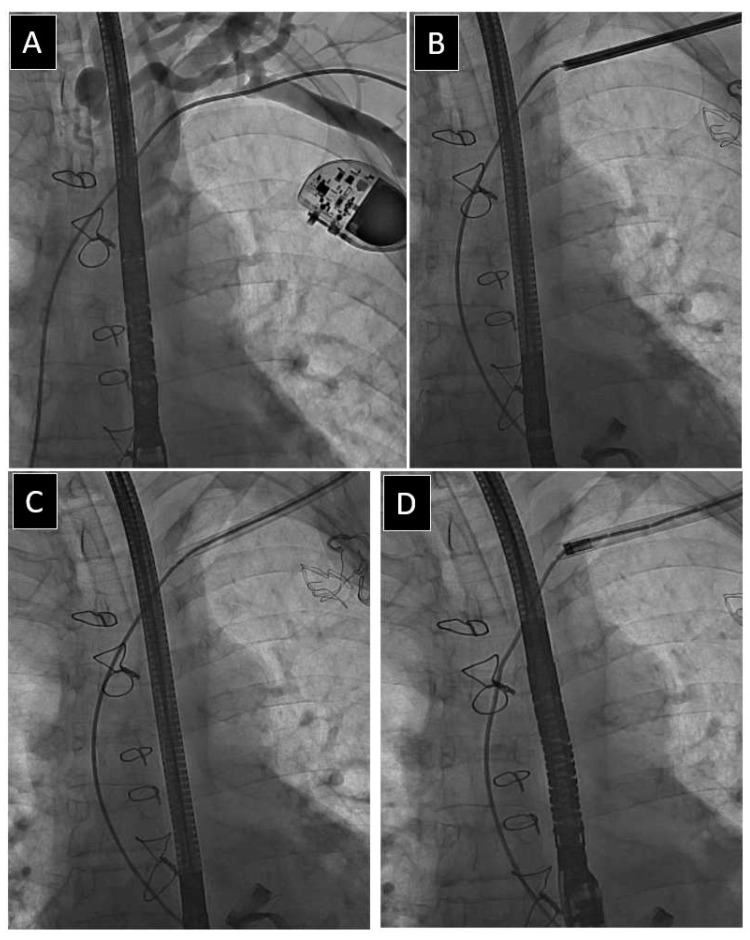
(**A**–**D**) Blockage in lead implant vein. Various tools (a polypropylene catheter, metal sheath, and Evolution) were used alternately to overcome resistance in the subclavian vein.

Blockage in the lead implant vein/subclavian region is caused by too parasternal a puncture of the subclavian vein or occlusion of the vein with dense scar tissue (Figure 4). This problem was present in 297 patients (7.98%). It was not associated with patient-related risk factors, only slightly associated with the number of leads and the presence of abandoned leads, but it was strongly related to implant duration. In our estimation, this is a UPD caused by a previous operator. Its presence prolonged lead extraction and forced the extractor to use second-line tools i.e., metal sheaths or mechanical rotational threaded sheaths when a standard stepwise and cross-over strategy was utilised. Furthermore, it was weakly related to major complications (MC), lower rates of radiographic success (non-removable lead fragments), and therefore lower rates of procedural success, but not to long-term mortality.

**Figure 5:**
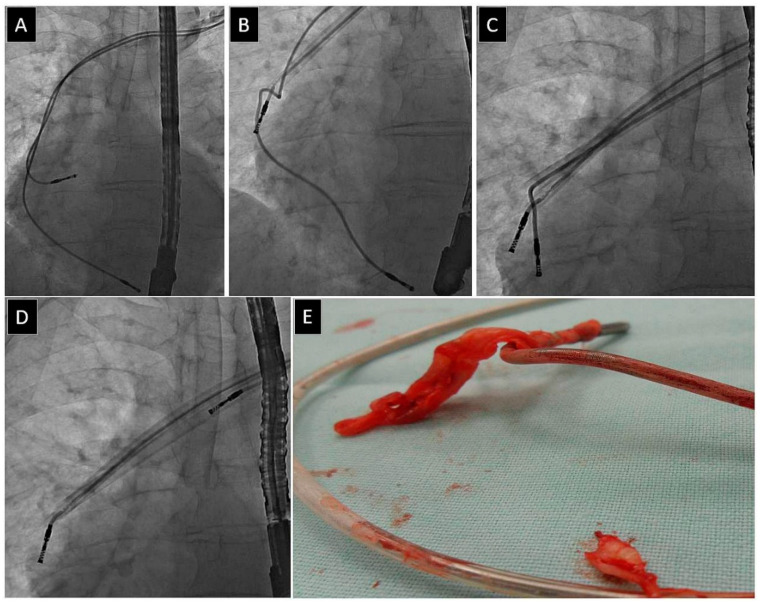
Lead-on-lead binding. The strong fibrous encapsulation prevents further advancement of the wire to free the lead (**A**,**B**). Sometimes it is necessary to use two polypropylene sheaths, which act together as scissors to separate the leads (**C**,**D**). Lead removed from the circulatory system, connective tissue remnants (**E**).

Lead-on-lead binding was the second most common UPD, as it occurred in 242 patients (6.59%). It was not associated with patient-related risk factors, weakly related to the number of leads (and leads being extracted), passive leads and abandoned lead presence and extraction, but significantly related to implant duration. Lead-on-lead binding prolonged lead extraction and forced the extractor to use different tips and tricks to free the targeted leads (Figure 5). It was strongly related to major complications (MC), more common emergent surgical interventions, lower rates of radiographic success (non-removable lead fragments), and therefore lower rates of procedural success, but not to long-term mortality. 

Lead fracture was the third most frequent UPD, as it occurred in 143 patients (3.84%). It was strongly dependent on patient-related risk factors (young age, higher EF, lower Charlson comorbidity index), strongly related to the presence of abandoned leads and the number of earlier CIED-related procedures, and the presence of abnormally long lead loops in the heart, strongly related to implant duration and the number of leads being extracted (all risk factors for the formation of fibrous tissue around the leads). It significantly prolonged the duration of lead extraction and forced the extractor to use second-line tools such as lassos, snares, basket catheters, an alternative venous approach (femoral, jugular, or combined). Furthermore, it was strongly associated with major complications (MC), more common emergent surgical interventions, lower rates of radiographic success (non-removable lead fragments), and therefore lower rates of procedural success, but not with long-term mortality.

Byrd dilator collapse/fracture was the fourth most common UPD, as it occurred in 127 patients (3.41%). The setting and consequences are similar to the fracture of the targeted lead described above. Byrd dilator collapse/fracture was described in detail in our previous study [22].

**Figure 6:**
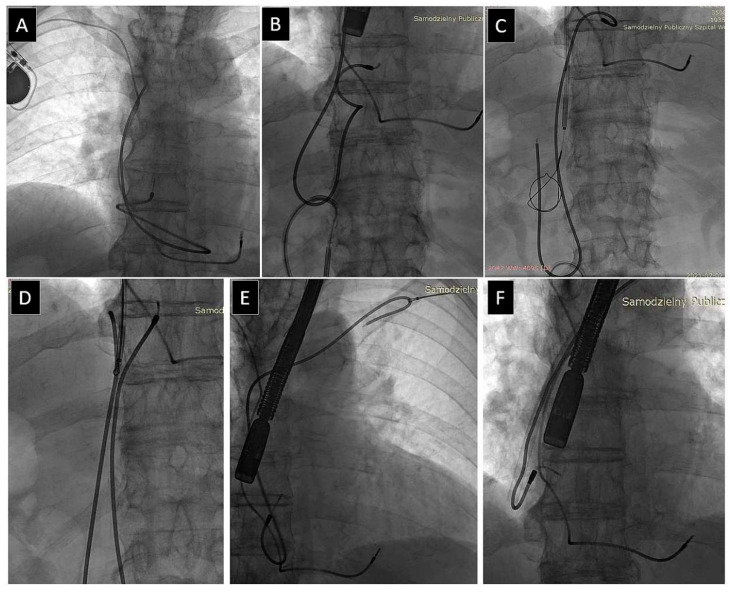
Use of alternative venous approach. A lead with its proximal end outside the generator pocket (**A**). If it is not retrievable with the lasso catheter via the superior approach, it has to be pulled down to free the end (**B**,**C**). The free end can be grasped again via the superior approach (**D**). The manoeuvre allows the operator to continue the procedure using conventional techniques (**E**,**F**) [32,41].

The use of an alternative venous approach was the fifth most frequent UPD, as it was seen in 122 patients (3.28%). We analysed all situations in which the lead implant venous access site was insufficient to complete the procedure (Figure 6). Similar to the fracture of the targeted lead and Byrd dilator collapse/fracture, this UPD occurred in patients who were younger at system implantation, with better health and a lower Charlson comorbidity index. Its occurrence was significantly related to the number of leads, the presence (and extraction) of abandoned leads, the number of previous CIED-related procedures, the presence of abnormally long lead loops in the heart, the extraction of passive leads, and, perhaps more than the others, lead dwell time. Finally, it was associated with the increased risk of MC and the need for rescue surgical intervention. However, this does not mean that one is a direct consequence of the other. Rather, both problems have the same cause, namely the lead dwell time.

Dislodgement of functional lead was the sixth most common UPD which occurred in 34 patients (0.91%). Except the left ventricular and His bundle pacing leads, we did not attach much importance to this UPD, but we entered the event in our database. It was not dependent on patient-related risk factors, slightly related to the number of leads and the presence of abandoned leads, but strongly related to lead dwell time. It slightly prolonged the duration of lead extraction, and it did not influence major complications (MC), the procedural success rate, or long-term mortality.

**Figure 7:**
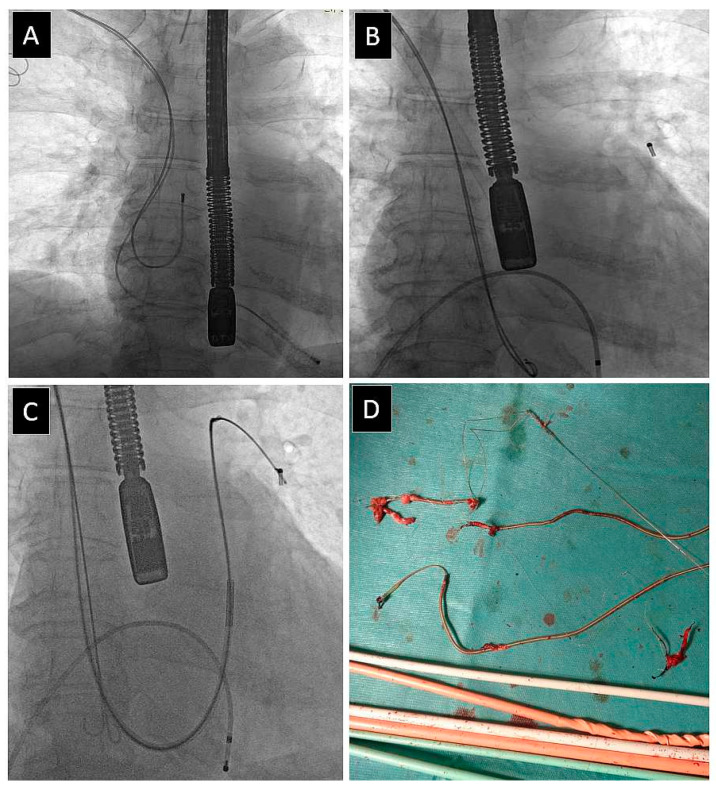
Loss of broken lead fragment. Image before the start of the TLE (**A**). The distal lead fragment detached during the lead dissection may flow in the bloodstream into the pulmonary circulation (**B**). It can usually be captured with a lasso catheter (**C**) and removed from the circulatory system (**D**) [38].

The loss of a broken lead fragment was the seventh most frequent UPD, which occurred in 22 patients (0.60%). All freed lead fragments were grasped and removed, but this event prolonged the duration of the procedure (Figure 7). It was not dependent on patient-related risk factors, strongly related to the number of leads, presence, and extraction of abandoned leads, significantly related to lead dwell time, and the extraction of passive leads. It significantly prolonged lead extraction and forced the operator to use second-line tools such as lassos, snares, basket catheters, and the alternative venous approach (femoral, jugular, combined). Its occurrence increased rates of major complications (MC), rescue surgical intervention, and decreased the procedural success rate without influencing long-term mortality.

## 4. Discussion

In this study, unexpected procedure difficulties occurred in 687 patients (18.46%), with a single UPD in 454 patients (12.20%) and multiple UPDs in 233 patients (6.26%). All UPDs as specific traps of TLE may prolong the procedure, but should not influence the final effect (clinical and procedural success and major complications). Thus, the proper identification of procedural complexity may influence the patient management strategy (referral of potentially difficult patients to experienced high-volume centres). Therefore, we tried to predict the occurrence of UPDs using popular calculators of TLE-related risk (SAFeTY-TLE, EROS) [7,8], and scores of procedure complexity (LED score, MB score, Advanced Lead Extraction score, and our recently elaborated LECOM score) [42,43,44,45].

It seems that increased TLE complexity did not influence long-term survival. For mortality after TLE, other factors such as general health status and the necessity of device therapy plays a predominant role [46].

The difficulty of lead extraction refers to the impossibility of sheath advancement, necessitating the use of more effective tools (using a stepwise approach and a cross-over strategy), starting from non-powered mechanical telescoping polypropylene sheaths (in various sizes) to powered mechanical sheaths (in various sizes) or laser sheaths, and the femoral approach [1,2,3,4,18]. There may also be an unexpected procedure difficulty (UPD), leading to embarrassing situations that must be solved to successfully complete the procedure. Such problems have been described in numerous case reports (the fracture of targeted leads [25,26,27,28,29,30,31,32,33,34], lead-on-lead binding [23,24,25,26,27], the use of an alternative venous approach [16,21,24,25,26,27,28,29,30,31,32,34], the loss of a broken lead fragment [37,38,39]), or in bigger reports (Byrd dilator collapse) [22]. The recently proposed LECOM calculator seems to be of practical use [42].

Our study mainly concerned the complexity of the TLE procedure, in particular the occurrence of unexpected difficulties that, on the one hand, prolong the procedure and increase its difficulty, and on the other hand (solved/overcome) cannot be classified as strictly defined complications. The impact of major complications on long-term survival has been the subject of previous [47] and recent reports [48]. They show that the mortality of patients after TLE is determined by the general health condition, but above all by the indications, the most important of which seems to be the extent and advancement of CIED infection. The results of our research correspond well in this aspect with the reports of Gomes et al. [47] and Arabia et al. [48]. A difficult, complex, but uncomplicated TLE procedure does not have a negative impact on long-term survival.

## 5. Conclusions

1. The difficulty of lead extraction refers not only to prolonged procedure time (fluoroscopy time), but sometimes to the necessity of solving a number of lesser-known unexpected procedure difficulties (UPDs).
2. UPDs are present in nearly one fifth of TLE procedures, and can occur simultaneously.
3. Unexpected procedure difficulties, which usually force the operator to expand the range of techniques and tools, should be considered as part of the learning and training in lead removal.

### Study Limitations

One weakness of this study is the fact that it presents the experience of the same team and the same first operator at three facilities. Therefore, the outcomes of the extraction may not represent the overall safety and efficacy of the transvenous extraction of leads with long implant durations. The data was collected on a systematic and ongoing basis, but was analysed retrospectively. All procedures were performed using all types of mechanical systems, but not laser powered sheaths. 

## Figures and Tables

**Table 1:** TLE procedure indications, including goal and type of procedure.

Main/Predominant Indication for TLE	N	%
Systemic infection	815	21.90%
Local (pocket) infection	357	9.59%
Mechanical lead damage (electric failure)	1002	26.93%
Lead dysfunction (exit/entry block, extracardiac pacing)	839	22.55%
Change of pacing mode/upgrading, downgrading	233	6.26%
Abandoned Lead/prevention of abandonment (AF, overmuch of leads)	102	2.74%
Another noninfective indication: threatener/potentially threatener lead (loops, free ending, left heart), MRI indication, cancer, pain of pocket, disappearance of indication for continuation of pacing/ICD	241	6.48%
Recapturing venous access (symptomatic occlusion, SVC syndrome, lead replacement/upgrading)	132	3.55%
All TLE procedures	3721	100.00%
Goal of TLE		
System removal-infection	1172	31.49%
Up-grading	449	12.07%
Down-grading	160	4.30%
Lead replacement	1742	46.82%
Superfluous lead extraction	29	0.78%
Redundant whole system removal	131	3.52%
System removal-re-implantation deferred	38	1.02%
All TLE procedures	3721	100.00%

TLE—transvenous lead extraction, AF—atrial fibrillation, MRI—magnetic resonance imaging, ICD—implantable cardioverter defibrillator, SVC—superior vena cava.

**Table 2:** Potential patient-related risk factors for the occurrence of unexpected procedure difficulties.

Potential Patient-Related Risk Factors for the Occurrence of UPD Related to System and History of Pacing								
	Number of Patients/Events *	Patient Age during TLE [Years]	Patient Age at First System Implantation [Years]	Female Gender	Ischemic Heart Disease Aetiology	LVEF [%]	Charlson’s Co-Morbidity Index [Points]	Infectious Indications for TLE (All)
		Mean ± SD	Mean ± SD	N (%)	N (%)	Mean ± SD	Mean ± SD	N (%)
All patients	3721 (100.0)	66.01 ± 15.63	57.55 ± 17.11	1419 (38.14)	2077 (55.82)	49.44 ± 13.28	4.72 ± 3.37	1176 (31.60)
Trouble-free lead extraction	3034 (81.53)	66.68 ± 15.07	59.08 ± 16.20	1157 (38.13)	1733 (57.12)	48.85 ± 15.50	4.83 ± 3.67	997 (32.86)
Blockage in lead implant vein/subclavian region *	297 (7.98)	63.41 ± 17.80 *p* = 0.007	51.05 ± 19.12 *p* = 0.001	102 (34.34) *p* = 0.096	171 (57.58) *p* = 0.719	51.45 ± 14.38 *p* = 0.045	4.67 ± 3.92 *p* = 0.180	63 (21.21) *p* = 0.001
Byrd dilatator collapse/fracture *	127 (3.41)	58.04 ± 19.98 *p* = 0.001	44.48 ± 21.66 *p* = 0.001	55 (43.31) *p* = 0.306	53 (41.73) *p* = 0.007	52.70 ± 14.11 *p* = 0.010	3.51 ± 3.77 *p* = 0.001	34 (26.77) *p* = 0.121
Lead-on-lead binding *	242 (6.59)	65.82 ± 14.53 *p* = 0.423	52.90 ± 17.09 *p* = 0.001	103 (42.56) *p* = 0.174	134 (55.37) *p* = 0.836	51.69 ± 14.23 *p* = 0.022	4.29 ± 3.35 *p* = 0.074	70 (28.93) *p* = 0.191
Fracture of targeted lead *	143 (3.84)	58.7 8 ± 19.01 *p* = 0.001	43.25 ± 20.24 P=0.001	61 (42.66) P=0.201	52 (36.36) *p* = 0.001	55.05 ± 11.74 *p* = 0.001	3.59 ± 3.73 *p* = 0.001	42 (29.37) *p* = 0.950
Use of alternative venous approach *	122 (3.28)	63.25 ± 16.30 *p* = 0.042	49.80 ± 18.48 *p* = 0.001	42 (34.43) *p* = 0.685	44 (36.07) *p* = 0.001	53.41 ± 13.61 *p* = 0.002	3.78 ± 3.40 *p* = 0.003	53 (43.44) *p* = 0.009
Loss of broken lead fragment *	22 (0.60)	67.68 ± 13.66 *p* = 0.988	51.73 ± 18.65 *p* = 0.251	8 (36.36) *p* = 0.640	9 (40.91) *p* = 0.333	53.05 ± 12.47 *p* = 0.333	4.27 ± 3.34 *p* = 0.673	7 (31.82) *p* = 0.570
Functional lead dislodgement *	34 (0.91)	71.12 ± 12.56 *p* = 0.064	63.91 ± 14.49 *p* = 0.020	14 (41.18) *p* = 0.868	32 (94.12) *p* = 0.001	49.62 ± 16.04 *p* = 0.958	5.11 ± 3.17 *p* = 0.207	5 (14.71) *p* = 0.049
Procedures with one UPD	454 (12.20)	63.36 ± 17.83 *p* = 0.001	52.29 ± 19.04 *p* = 0.001	170 (37.45) *p* = 0.889	394 (86.78) *p* = 0.041	51.30 ± 14.49 *p* = 0.006	4.27 ± 3.20 *p* = 0.001	111 (24.45) *p* = 0.001
Procedures with multiple UPDs	233 (6.26)	62.34 ± 17.21 *p* = 0.001	48.17 ± 19.48 *p* = 0.001	92 (39.49) *p* = 0.858	109 (46.78) *p* = 0.007	53.27 ± 13.67 *p* = 0.001	4.12 ± 3.75 *p* = 0.001	68 (29.19) *p* = 0.001
Procedures with any UPD	687 (18.43)	63.02 ± 17.61 *p* = 0.001	50.89 ± 19.83 *p* = 0.001	262 (38.14) *p* = 0.988	344 (50.07) *p* = 0.002	51.94 ± 14.24 *p* = 0.001	4.22 ± 3.66 *p* = 0.001	179 (26.06) *p* = 0.001

* As unexpected procedure difficulty could occur more than once in the same patient, the total number of UPDs does not correspond to the number of extraction procedures. TLE—transvenous lead extraction, UPD—unexpected procedure difficulty, LVEF— left ventricular ejection fraction.

**Table 3:** Potential risk factors for the occurrence of unexpected procedure difficulties related to system, history of pacing, and extraction procedure.

Potential Risk Factors for the Occurrence of UPD Related to System and History of Pacing								
	Number of Patients/Events *	Number of Leads in the Heart before TLE	≥4 Leads in the Heart before TLE	Abandoned Lead before TLE	Leads on Both Sides of the Chest before TLE	Number of Procedures before Lead Extraction	Abnormally Long Lead Loop in the Atrium before TLE	Abnormally Long Lead Loop in the Ventricle before TLE
	N (%)	Mean ± SD	N (%)	N (%)	N (%)	Mean ± SD	N (%)	N (%)
All patients	3721 (100.0)	1.95 ± 0.74	117 (3.14)	413 (11.10)	106 (2.85)	1.86 ± 1.08	361 (9.70)	179 (4.81)
Trouble-free lead extraction	3034 (81.53)	1.90 ± 0.71	66 (2.17)	254 (8.37)	51 (1.68)	1.72 ± 0.99	244 (8.04)	105 (3.46)
Blockage in lead implant vein/subclavian region *	297 (7.98)	2.07 ± 0.81 *p* = 0.001	17 (5.72) *p* = 0.016	47 (15.83) *p* = 0.004	13 (4.38) *p* = 0.001	2.26 ± 1.27 *p* = 0.001	36 (12.12) *p* = 0.017	14 (4.71) *p* = 0. 206
Byrd dilator collapse/fracture *	127 (3.41)	2.05 ± 0.84 *p* = 0.405	9 (7.08) *p* = 0.022	28 (22.05) *p* = 0.001	14 (11.02) *p* = 0.001	2.35 ± 1.33 *p* = 0.001	20 (15.75) *p* = 0.015	9 (7.09) *p* = 0.300
Lead-on-lead binding *	242 (6.59)	2.46 ± 0.72 *p* = 0.001	21 (8.68) *p* = 0.001	72 (29.75) *p* = 0.001	24 (9.92) *p* = 0.001	2.49 ± 1.28 *p* = 0.001	42 (17.36) *p* = 0.001	30 (12.40) *p* = 0.001
Fracture of targeted lead *	143 (3.84)	2.17 ± 0.85 *p* = 0.003	13 (9.09) *p* = 0.001	43 (30.07) *p* = 0.001	10 (6.99) *p* = 0.001	2.73 ± 1.40 *p* = 0.001	28 (19.58) *p* = 0.001	21 (14.68) *p* = 0.001
Use of alternative venous approach *	122 (3.28)	2.66 ± 1.07 *p* = 0.001	28 (22.95) *p* = 0.001	69 (56.56) *p* = 0.001	36 (29.51) *p* = 0.001	3.05 ± 1.63 *p* = 0.001	38 (31.15) *p* = 0.001	40 (32.78) *p* = 0.001
Loss of broken lead fragment *	22 (0.60)	2.64 ± 1.14 *p* = 0.038	6 (27.27) *p* = 0.0906	10 (45.45) *p* = 0.001	6 (27.27) *p* = 0.003	2.52 ± 1.03 *p* = 0.007	4 (18.18) *p* = 0.969	8 (36.36) *p* = 0.001
Functional lead dislodgement *	34 (0.91)	2.38 ± 0.65 *p* = 0.001	2 (5.88) *p* = 0.666	6 (17.65) *p* = 0.322	1 (2.94) *p* = 0.630	2.06 ± 1.13 *p* = 0.396	5 (14.71) *p* = 0.398	4 (11.77) *p* = 0.198
Procedures with one technical problem	454 (12.20)	2.08 ± 0.79 *p* = 0.001	21 (4.63) *p* = 0.009	79 (17.04) *p* = 0.001	23 (5.07) *p* = 0.001	2.19 ± 1.16 *p* = 0.001	71 (15.64) *p* = 0.001	39 (8.59) *p* = 0.001
Procedures with multiple UPDs	233 (6.26)	2.39 ± 0.88 *p* = 0.001	30 (12.88) *p* = 0.001	80 (34.34) *p* = 0.001	32 (13.73) *p* = 0.001	2.74 ± 1.50 *p* = 0.001	46 (19.74) *p* = 0.001	35 (15.02) *p* = 0.001
Procedures with any UPD	687 (18.43)	2.18 ± 0.84 *p* = 0.001	51 (7.42) *p* = 0.001	159 (23.14) *p* = 0.001	55 (8.01) *p* = 0.001	2.37 ± 1.30 *p* = 0.001	117 (17.03) *p* = 0.001	74 (10.77) *p* = 0.001
Potential TLE-related risk factors for the occurrence of UPD during lead extraction								
	Number of patients / events*	Number of extracted leads per patient	Three or more leads extracted	Extraction of defibrillation lead(s)	Extraction of abandoned lead(s) (any)	Extraction of passive fixation lead(s) (excluding CS lead)	Oldest extracted lead [months]	Cumulative dwell time of extracted leads [years]
	N (%)	mean ± SD	N (%)	N (%)	N (%)	N (%)	mean ± SD	mean ± SD
All patients	3721 (100.0)	1.65 ± 0.73	390 (10.48)	1021 (27.44)	379 (10.19)	2186 (80.34)	100.3 ± 75.23	13.71 ± 12.67
Trouble-free lead extraction	3034 (81.53)	1.59 ± 0.68	263 (8.67)	877 (28.91)	228 (7.51)	1666 (54.91)	89.86 ± 68.49	11.82 ± 10.89
Blockage in lead implant vein/subclavian region *	297 (7.98)	1.86 ± 0.83 *p =* 0.001	52 (17.51) *p* = 0.001	63 (21.21 *p* = 0.015	45 (15.15) *p* = 0.004	205 (69.02) *p* = 0.013	149.3 ± 93.17 *p* = 0.001	22.19 ± 17.75 *p* = 0.001
Byrd dilator collapse/fracture *	127 (3.41)	1.85 ± 0.83 *p* = =0.010	23 (18.11) *p* = 0.003	42 (33.07) *p* = 0.122	25 (19.69) *p* = 0.001	93 (73.23) *p* = 0.022	158.9 ± 87.99 *p* = 0.001	23.65 ± 19.06 *p* = 0.001
Lead-on-lead binding *	242 (6.59)	2.28 ± 0.72 *p* = 0.001	69 (28.51) *p* = 0.001	43 (17.77) *p* < 0.001	68 (28.10) *p* = 0.001	191 (78.93) *p* = 0.001	155.2 ± 89.70 *p* = 0.001	27.67 ± 18.44 *p* = 0.001
Fracture of targeted lead *	143 (3.84)	2.04 ± 0.88 *p* = 0.001	34 (23.78) *p* = 0.001	13 (28.67) *p* < 0.001	41 (28.67) *p* = 0.001	134 (93.71) *p* = 0.001	185.5 ± 85.97 *p* = 0.001	28.53 ± 16.99 *p* = 0.001
Use of alternative venous approach *	122 (3.28)	2.27 ± 1.15 *p* = 0.001	45 (36.86) *p* = 0.001	12 (54.92) *p* < 0.001	67 (54.92) *p* = 0.001	117 (95.90) *p* = 0.001	161.0 ± 83.89 *p* = 0.001	26.50 ± 18.45 *p* = 0.001
Loss of broken lead fragment *	22 (0.60)	2.41 ± 1.01 *p* = 0.001	9 (40.91) *p* = 0.003	1 (4.55) *p* = 0.030	10 (45.46) *p* = 0.001	21 (94.46) *p* = 0.051	192.2 ± 109.6 *p* = 0.001	34.61 ± 20.63 *p* = 0.001
Functional lead dislodgement *	34 (0.91)	2.03 ± 0.58 *p* = 0.001	4 (11.77) *p* = 0.946	8 (17.65) *p* = 0.749	6 (17.65) *p* = 0.241	26 (76.47) *p* = 0.130	86.53 ± 58.69 *p* = 0.400	13.82 ± 10.98 *p* = 0.422
Procedures with one UPD	454 (12.20)	1.77 ± 0.81 *p* = 0.001	54 (11.89) *p* = 0.030	104 (22.91) *p* < 0.001	73 (16.08) *p* = 0.001	319 (70.26) *p* = 0.001	132.8 ± 79.20 *p* = 0.001	18.55 ± 13.03 *p* = 0.001
Procedures with multiple UPDs	233 (6.26)	2.24 ± 0.65 *p* = 0.001	73 (31.33) *p* = 0.001	40 (33.48) *p* < 0.001	78 (33.48) *p* = 0.001	201 (86.27) *p* = 0.001	170.5 ± 92.58 *p* = 0.001	28.79 ± 19.36 *p* = 0.001
Procedures with any UPD	687 (18.43)	1.93 ± 0.85 *p* = 0.001	127 (18.49) *p* = 0.001	144 20.96) *p* < 0.001	151 (21.98) *p* = 0.001	520 (75.69) *p* = 0.001	145.6 ± 85.78 *p* = 0.001	22.02 ± 16.20 *p* = 0.001

* As unexpected procedure difficulty could occur more than once in the same patient, the total number of UPDs does not correspond to the number of extraction procedures. TLE—transvenous lead extraction, UPD—unexpected procedure difficulty, ICD—implantable cardioverter defibrillator, CS—coronary sinus.

**Table 4:** TLE-related risk, complexity indicators and risk calculators.

	Number of Patients/Events *	Lead Extraction Time (Sheath-to-Sheath) [Minutes]	Average Time of Single Lead Extraction [Minutes]	Use of Evolution (Old or New) or TightRail	Use of Metal Sheaths	Use of Lasso Catheters/Snares	Use of Basket Catheters
		Mean ± SD	Mean ± SD	N (%)	N (%)	N (%)	N (%)
Procedure Risk and Complexity Indicators							
All patients	3721 (100.0)	14.98 ± 22.67	8.89 ± 12.35	55 (1.48)	299 (8.04)	139 (3.74)	41 (1.10)
Trouble-free lead extraction	3034 (81.53)	9.94 ± 10.96	6.35 ± 6.32	8 (0.26)	1 (0.03)	14 (0.46)	4 (0.13)
Blockage in lead implant vein/subclavian region *	297 (7.98)	32.63 ± 36.89 *p =* 0.001	17.26 ± 18.36 *p =* 0.001	22 (7.41) *p =* 0.001	297 (100.0) *p =* 0.001	36 (12.12) *p =* 0.001	4 (1.35) *p =* 0. 831
Byrd dilator collapse/fracture *	127 (3.41)	41.89 ± 47.82 *p =* 0.001	22.34 ± 24.41 *p =* 0.001	21 (16.54) *p =* 0.001	31 (24.41) *p =* 0.001	22 (17.32) *p =* 0.001	2 (1.57) *p =* 0.501
Lead-on-lead binding *	242 (6.59)	41.56 ± 47.84 *p =* 0.001	17.45 ± 18.26 *p =* 0.001	25 (10.33) *p =* 0.001	64 (26.45) *p =* 0.001	38 (15.70) *p =* 0.001	3 (1.24) *p =* 0.828
Fracture of targeted lead *	143 (3.84)	62.36 ± 52.38 *p =* 0.001	33.65 ± 32.26 *p =* 0.001	20 (13.99) *p =* 0.001	40 (27.97) *p =* 0.001	97 (67.83) *p =* 0.001	10 (6.99) *p =* 0.001
Use of alternative venous approach *	122 (3.28)	79.88 ± 56.65 *p =* 0.001	40.81 ± 33.33 *p =* 0.001	7 (5.74) *p =* 0.001	24 (19.67) *p =* 0.001	50 (40.98) *p =* 0.001	33 (27.05) *p =* 0.001
Loss of broken lead fragment *	22 (0.60)	97.27 ± 80.85 *p =* 0.001	45.45 ± 49.44 *p =* 0.001	3 (13.64) *p =* 0.001	6 (27.27) *p =* 0.059	18 (81.82) *p =* 0.001	3 (13.64) *p =* 0.001
Functional lead dislodgement *	34 (0.91)	28.18 ± 33.27 *p =* 0.001	13.51 ± 14.24 *p =* 0.003	0 (0.00) *p =* 0.990	2 (5.88) *p =* 0.868	1 (2.94) *p =* 0.885	3 (8.82) *p =* 0.002
Procedures with one UPD	454 (12.20)	24.67 ± 25.04 *p =* 0.001	14.99 ± 15.80 *p =* 0.001	14 (3.08) *p =* 0.001	183 (40.31) *p =* 0.001	47 (10.35) *p =* 0.001	21 (4.63) *p =* 0.001
Procedures with multiple UPDs	233 (6.26)	60.83 ± 52.96 *p =* 0.001	29.23 ± 25.23 *p =* 0.001	33 (14.16) *p =* 0.001	115 (49.36) *p =* 0.001	78 (33.48) *p =* 0.001	16 (6.87) *p =* 0.001
Procedures with any UPD	687 (18.43)	36.94 ± 40.69 *p =* 0.001	19.81 ± 22.33 *p =* 0.001	47 (6.84) *p =* 0.001	298 (43.38) *p =* 0.001	125 (18.20) *p =* 0.001	37 (5.39) *p =* 0.001
TLE-related risk, procedure complexity, and calculators for prediction of UPDs							
	Number of patients/UPD *	SAFeTY-TLE risk of MC [%]	EROS score 2 or 3 points	LECOM score [%]	MBscore [points]	LEDscore [points]	Advanced TLE (Mazzone) score [points]
	N (%)	mean ± SD	N (%)	mean ± SD	mean ± SD	mean ± SD	mean ± SD
All patients	3721 (100.0)	1.72 ± 2.98	1398 (37.57)	20.94 ± 18.95	2.58 ± 1.25	10.04 ± 6.40	2.14 ± 0.92
Trouble-free lead extraction	3034 (81.53)	1.40 ± 2.31	1067 (35.16)	18.12 ± 16.46	2.43 ± 1.25	9.11 ± 5.81	2.07 ± 0.94
Blockage in lead implant vein/subclavian region *	297 (7.98)	2.93 ± 4.82 *p =* 0.001	149 (50.17) *p =* 0.001	30.33 ± 22.48 *p =* 0.001	3.15 ± 1.04 *p =* 0.001	14.30 ± 7.92 *p =* 0.001	2.40 ± 0.82 *p =* 0.001
Byrd dilator collapse/fracture *	127 (3.41)	3.71 ± 6.51 *p =* 0.001	70 (55.12) *p =* 0.001	34.42 ± 23.52 *p =* 0.001	3.32 ± 0.85 *p =* 0.001	15.20 ± 7.53 *p =* 0.001	2.63 ± 0.73 *p =* 0.001
Lead-on-lead binding *	242 (6.59)	3.84 ± 5.86 *p =* 0.001	114 (47.11) *p =* 0.001	38.15 ± 24.36 *p =* 0.001	3.55 ± 0.84 *p =* 0.001	15.16 ± 7.61 *p =* 0.001	2.63 ± 0.74 *p =* 0.001
Targeted lead fracture*	143 (3.84)	4.47 ± 6.54 *p =* 0.001	84 (58.74) *p =* 0.001	43.86 ± 22.64 *p =* 0.001	3.54 ± 0.73 *p =* 0.001	17.41 ± 7.21 *p =* 0.001	2.54 ± 0.71 *p =* 0.001
Use of alternative venous approach *	122 (3.28)	4.81 ± 6.43 *p =* 0.001	65 (53.28) *p =* 0.001	48.37 ± 27.23 *p =* 0.001	3.33 ± 1.01 *p =* 0.001	15.48 ± 7.15 *p =* 0.001	2.39 ± 0.84 *p =* 0.001
Loss of broken lead fragment*	22 (0.60)	3.57 ± 3.18 *p =* 0.012	14 (65.64) *p =* 0.021	40.64 ± 29.46 *p =* 0.001	3.46 ± 0.96 *p =* 0.001	18.36 ± 9.40 *p =* 0.005	2.46 ± 0.91 *p =* 0.001
Functional lead dislodgement *	34 (0.91)	1.52 ± 2.08 *p =* 0.866	6 (17.67) *p =* 0.001	23.67 ± 21.53 *p =* 0.222	2.85 ± 1.02 *p =* 0.287	9.41 ± 5.09 *p =* 0.814	2.38 ± 0.78 *p =* 0.180
Procedures with one UPD	454 (12.20)	2.53 ± 3.45 *p =* 0.001	209 (46.04) *p =* 0.001	28.60 ± 20.47 *p =* 0.001	3.05 ± 1.07 *p =* 0.001	12.86 ± 6.64 *p =* 0.001	2.34 ± 0.82 *p =* 0.001
Procedures with multiple UPDs	233 (6.26)	4.35 ± 6.30 *p =* 0.001	122 (52.36) *p =* 0.001	42.36 ± 25.46 *p =* 0.001	3.38 ± 0.73 *p =* 0.001	16.40 ± 7.81 *p =* 0.001	2.65 ± 0.72 *p =* 0.001
Procedures with any UPD	687 (18.43)	3.14 ± 4.69 *p =* 0.001	331 (48.18) *p =* 0.001	33.22 ± 23.18 *p =* 0.001	3.23 ± 1.00 *p =* 0.001	14.06 ± 7.25 *p =* 0.001	2.45 ± 0.80 *p* = 0.001

* As unexpected procedure difficulty could occur more than once in the same patient, the total number of UPDs does not correspond to the number of extraction procedures. Average time of single lead extraction—sheath-to-sheath time [minutes] divided by the number of extracted leads, TLE—transvenous lead extraction, UPD—unexpected procedure difficulty; SAFeTY-TLE risk score of major complications—risk of MC in %; EROS score 2 or 3—increased risk of significant procedural complications that require urgent surgical intervention (1–3); LECOM score—combined: lead dilatation time, use of second-line or advanced tools and advanced techniques; MB score—the need for advanced tools to achieve TLE success; LED score—difficult TLE, defined by means of fluoroscopy time; Advanced Lead Extraction (Mazzone) score—the need for use of advanced TLE techniques.

**Table 5:** Major complications of lead extraction, effectiveness, and short-, mid- and long-term mortality.

TLE Outcome (Major Complications, Effectiveness, and Mortality)							
	Number of Patients/Events *	Major Complications (Any)	Haemoperi-Cardium	Haemo-Thorax	Tricuspid Valve Damage during TLE (Severe)	Emergent Surgical Intervention	Partial Radiographic Success
	N (%)	N (%)	N (%)	N (%)	N (%)	N (%)	N (%)
All patients	3721 (100.0)	76 (2.04)	45 (1.29)	5 (0.13)	22 (0.59)	42 (1.13)	147 (3.95)
Trouble-free lead extraction	3034 (81.53)	39 (1.29)	21 (0.69)	3 (0.10)	11 (0.36)	19 (0.63)	58 (1.91)
Blockage in lead implant vein/subclavian region *	297 (7.98)	12 (4.04) *p =* 0.004	8 (2.69) *p =* 0.008	0 (0.00) *p =* 0. 878	3 (1.01) *p =* 0.577	6 (2.02) *p =* 0.433	35 (11.79) *p =* 0.001
Byrd dilator collapse/fracture *	127 (3.41)	9 (7.09) *p =* 0.001	5 (3.94) *p =* 0.009	0 (0.00) *p =* 0.421	5 (3.94) *p =* 0.001	4 (3.15) *p =* 0.318	18 (14.71) *p =* 0.001
Lead-on-lead binding *	242 (6.59)	25 (10.33) *p =* 0.001	18 (7.44) *p =* 0.001	1 (0.41) *p =* 0.753	6 (2.48) *p =* 0.001	17 (7.03) *p =* 0.001	25 (10.33) *p =* 0.001
Fracture of targeted lead *	143 (3.84)	12 (8.39) *p =* 0.001	8 (5.60) *p =* 0.001	1 (0.70) *p =* 0.001	4 (2.80) *p =* 0.473	7 (4.90) *p =* 0.001	61 (42.66) *p =* 0.001
Use of alternative venous approach *	122 (3.28)	6 (4.92) *p =* 0.001	5 (4.10) *p =* 0.008	0 (0.00) *p =* 0.414	1 (0.82) *p =* 0.721	4 (3.28) *p =* 0.085	26 (21.31) *p =* 0.001
Loss of broken lead fragment *	22 (0.60)	4 (18.18) *p =* 0.001	1 (4.55) *p =* 0.553	0 (0.00) *p =* 0.001	3 (13.64) *p =* 0.001	1 (4.55) *p =* 0.436	7 (31.82) *p =* 0.001
Functional lead dislodgement *	34 (0.91)	1 (2.94) *p =* 0.808	0 (0.00) *p =* 0.934	0 (0.00) *p =* 0.032	1 (2.94) *p =* 0.499	0 (0.00) *p =* 0.855	4 (11.77) *p =* 0.384
Procedures with one UPD	454 (12.20)	17 (3.74) *p =* 0.001	11 (2.42) *p =* 0.001	2 (0.44) *p =* 0.269	3 (0.66) *p =* 0.001	12 (2.64) *p =* 0.001	30 (6.61) *p =* 0.001
Procedures with multiple UPDs	233 (6.26)	20 (8.58) *p =* 0.001	13 (5.58) *p =* 0.001	0 (0.00) *p =* 0.517	8 (3.43) *p =* 0.001	11 (4.72) *p =* 0.001	59 (25.32) *p =* 0.001
Procedures with any UPD	687 (18.43)	37 (5.39) *p =* 0.001	24 (3.49) *p =* 0.001	2 (0.29) *p =* 0.517	11 (1.60) *p =* 0.001	23 (3.35) *p =* 0.001	89 (12.96) *p =* 0.001
TLE effectiveness, and short-, mid- and long-term mortality							
	Number of patients/events*	Complete procedural success	Death, procedure-related (intra-, post-procedural)	Death, indication-related (intra-, post-procedural	One month/30-day mortality (48 h–30 days)	1 year mortality after TLE (31-365 days)	All deaths during LT-FU (0 to last FU)
	N (%)	N (%)	N (%)	N (%)	N (%) Log rank P	N (%) Log rank P	N (%) Log rank P
All patients	3721 (100.0)	3549 (95.38)	6 (0.16)	4 (0.11)	63 (1.69)	250 (8.41)	1354 (36.39)
Trouble-free lead extraction	3034 (81.53)	2962 (97.63)	3 (0.10)	2 (0.07)	50 (1.65)	257 (8.47)	1134 (37.38)
Blockage in lead implant vein/subclavian region *	297 (7.98)	262 (88.22) *p =* 0.001	0 (0.00) *p =* 0.465	2 (0.67) *p =* 0.031	2 (0.67) *p =* 0. 346	14 (4.71) *p =* 0.096	72 (24.24) *p =* 0.073
Byrd dilator collapse/fracture*	127 (3.41)	106 (83.46) *p =* 0.001	0 (0.00) *p =* 0.643	0 (0.00) *p =* 0.321	3 (2.63) *p =* 0.187	7 (5.51) *p =* 0.876	34 (26.77) *p =* 0.271
Lead-on-lead binding *	242 (6.59)	208 (85.15) *p =* 0.001	3 (1.24) *p =* 0.008	0 (0.00) *p =* 0.628	4 (1.65) *p =* 0.309	11 (4.55) *p =* 0.463	82 (33.88) *p =* 0.315
Fracture of targeted lead *	143 (3.84)	76 (53.15) *p =* 0.001	0 (0.00) *p =* 0.565	0 (0.00) *p =* 0.368	2 (1.40) *p =* 0.281	12 (8.39) *p =* 0.201	48 (33.57) *p =* 0.241
Use of alternative venous approach *	122 (3.28)	91 (74.59) *p =* 0.001	1 (0.82) *p =* 0.504	0 (0.00) *p =* 0.708	2 (1.64) *p =* 0.207	15 (12.30) *p =* 0.013	59 (48.36) *p =* 0.762
Loss of broken lead fragment *	22 (0.60)	13 (59.09) *p =* 0.001	0 (0.00) *p =* 0.004	0 (0.00) *p =* 0.001	0 (0.00) *p =* 0.607	1 (4.55) *p =* 0.714	11 (50.00) *p =* 0.749
Functional lead dislodgement *	34 (0.91)	30 (88.24) *p =* 0.139	0 (0.00) *p =* 0.035	0 (0.00) *p =* 0.014	0 (0.00) *p =* 0.445	3 (8.82) *p =* 0.987	14 (41.18) *p =* 0.628
Procedures with one UPD	454 (12.20)	423 (93.17) *p =* 0.001	2 (0.44) *p =* 0.654	2 (0.44) *p =* 0.004	6 (1.32) *p =* 0.572	35 (7.71) *p =* 0.518	144 (31.72) *p =* 0.052
Procedures with multiple UPDs	233 (6.26)	164 (70.39) *p =* 0.001	1 (0.43) *p =* 0.806	0 (0.00) *p =* 0.097	7 (3.00) *p =* 0.119	21 (9.01) *p =* 0.737	77 (33.05) *p =* 0.137
Procedures with any UPD	687 (18.43)	587 (85.44) *p =* 0.001	3 (0.44) *p =* 0.093	2 (0.29) *p =* 0.025	13 (1.89) *p =* 0.676	56 (8.15) *p =* 0.737	220 (32.02) *p =* 0.019

* As unexpected procedure difficulty could occur more than once in the same patient, the total number of UPDs does not correspond to the number of extraction procedures. TLE—transvenous lead extraction, UPD—unexpected procedure difficulty.

**Table 6:** Prognostic factors for the occurrence of UPDs—results of stepwise multivariable regression analysis.

Prognostic Factors for the Occurrence of UPDs						
	Univariable Logistic Regression			Multivariable Regression		
	OR	95%CI	*p*	OR	95%CI	*p*
Procedures with one UPD						
Patient age at first system implantation [by 1 year]	0.979	0.973–0.984	*p* < 0.001	0.989	0.983–0.995	*p* < 0.001
Infectious indications for TLE (all) [y/n]	0.635	0.507–0.797	*p* < 0.001	0.667	0.526–0.845	*p* = 0.001
Number of leads in the heart before TLE	1.364	1.199–1.552	*p* < 0.001	1.350	1.178–1.546	*p* < 0.001
Abnormally long lead loop in the atrium before TLE [y/n]	2.032	1.519–2.720	*p* < 0.001	1.898	1.398–2.578	*p* < 0.001
Abnormally long lead loop in the ventricle before TLE [y/n]	2.787	1.950–3.982	*p* < 0.001	2.318	1.594–3.370	*p* < 0.001
Oldest extracted lead [by 1 year]	1.090	1.075–1.106	*p* < 0.001	1.071	1.054–1.088	*p* < 0.001
Procedures with multiple UPDs						
Patient age at first system implantation [by year]	0.970	0.963–0.976	*p* < 0.001	0.985	0.977–0.994	*p* = 0.001
Number of leads in the heart before TLE	2.181	1.851–2.570	*p* < 0.001	1.579	1.302–1.915	*p* < 0.001
Leads on both sides of the chest before [y/n]	9.625	6.072–15.30	*p* < 0.001	2.402	1.355–4.258	*p* = 0.003
Abnormally long lead loop in the atrium before TLE [y/n]	2.571	1.789–3.696	*p* < 0.001	2.214	1.484–3.304	*p* < 0.001
Abnormally long lead loop in the ventricle before TLE [y/n]	2.787	1.950–3.982	*p* < 0.001	3.208	2.056–5.005	*p* < 0.001
Extraction of passive fixation lead(s) (excluding CS lead) [y/n]	3.676	2.568–5.261	*p* < 0.001	1.613	1.088–2.393	*p* = 0.017
Oldest extracted lead [by 1 year]	1.137	1.117–1.158	*p* < 0.001	1.096	1.072–1.120	*p* < 0.001
Procedures with any UPD						
Patient age at first system implantation [by year]	0.975	0.971–0.979	*p* < 0.001	0.988	0.983–0.993	*p* < 0.001
Infectious indications for TLE (all) [y/n]	0.698	0.580–0.841	*p* < 0.001	0.685	0.558–0.841	*p* < 0.001
Number of leads in the heart before TLE	1.604	1.441–1.786	*p* < 0.001	1.395	1.230–1.582	*p* < 0.001
Leads on both sides of the chest before [y/n]	4.999	3.383–7.388	*p* < 0.001	2.017	1.270–3.203	*p* = 0.003
Abnormally long lead loop in the atrium before TLE [y/n]	2.208	1.728–2.821	*p* < 0.001	1.979	1.518–2.579	*p* < 0.001
Abnormally long lead loop in the ventricle before TLE [y/n]	3.517	2.622–4.717	*p* < 0.001	2.464	1.785–3.400	*p* < 0.001
Extraction of passive fixation lead(s) (excluding CS lead) [y/n]	2.293	1.896–2.773	*p* < 0.001	1.251	1.009–1.552	*p* = 0.041
Oldest extracted lead [by 1 year]	1.111	1.097–1.125	*p* < 0.001	1.080	1.064–1.096	*p* < 0.001

TLE—transvenous lead extraction, UPD—unexpected procedure difficulty, CS—coronary sinus.

## Data Availability

Readers can access the data supporting the conclusions of the study at www.usuwanieelektrod.pl (1 August 2022).
